# Clinical implications of primary “occult” vesicoureteral reflux in male children

**DOI:** 10.1007/s00330-024-10768-7

**Published:** 2024-04-22

**Authors:** Stefano Guarino, Anna Di Sessa, Giulio Rivetti, Giusy Capasso, Roberta Schiano di Cola, Antonietta Rimoli, Emanuele Miraglia del Giudice, Cesare Polito, Angela La Manna, Pierluigi Marzuillo

**Affiliations:** Department of Woman, Child and of General and Specialized Surgery, Università degli Studi della Campania “Luigi Vanvitelli”, Via Luigi De Crecchio 2, Naples, Italy

**Keywords:** Vesicoureteral reflux, Chronic kidney failure, Radiography, Radionuclide imaging, Urinary tract infection

## Abstract

**Objectives:**

To compare characteristics and outcomes of vesicoureteral reflux (VUR) detected solely on isotopic cystography (IC) (“occult” VUR) with voiding cystourethrography (VCUG)-detected VUR.

**Materials and methods:**

Between 2015 and 2020, we retrospectively enrolled all male children first undergoing VCUG and, if negative, IC in the same session. Kidney injury (KI) was defined by abnormal estimated glomerular filtration rate and/or blood pressure and/or proteinuria.

**Results:**

We enrolled 421 males with a median age of 3 months and a follow-up of 5.3 years. None exhibited KI initially, but 10% of those with VUR developed KI during follow-up. Two hundred and twenty-two patients (52.7%) did not show VUR, 152 (36.1%) had VCUG-diagnosed VUR, and 47 (11.2%) had occult VUR. Therefore, 47/199 patients (23.6%) with VUR had occult VUR. Among these, 34/47 (72.3%) had dilated VUR, and 22/47 (46.8%) exhibited split renal function < 45% and/or scar (scintigraphic damage). Compared to patients with occult VUR, those with VCUG-diagnosed VUR showed a similar prevalence of febrile urinary tract infection (fUTI) before and after VUR diagnostics and KI at the last follow-up but a higher prevalence of dilated VUR, of scintigraphic damage, and underwent surgery more frequently. At multiple logistic regression analysis, patients with VCUG-diagnosed VUR presented an increased risk of fUTI either before or after VUR diagnosis and of KI, while patients with occult VUR presented an increased risk of fUTI before (and among patients with dilated VUR also after) VUR diagnosis and of KI.

**Conclusion:**

Occult VUR affects 23.6% of male children with VUR with a non-negligible risk of VUR-associated KI and fUTI. IC could select, among males with recurrent fUTIs and negative VCUG, those requiring surgery for a possible dilated occult VUR.

**Clinical relevance statement:**

Vesicoureteral reflux may be overlooked in 25% of boys during VCUG, yet they are at risk of fUTIs and KI. In case of recurrent infections post-negative cystourethrography, IC could detect occult reflux, guiding surgical intervention.

## Key Points

- *VCUG exposes children to radiation and is currently the gold standard for diagnosing VUR.*
- *A quarter of male children with VUR were diagnosed by IC despite normal VCUG.*
- *Recurrent fUTIs after negative VCUG may benefit from evaluation with IC.*

## Introduction

The gold standard for diagnosing vesicoureteral reflux (VUR) is voiding cystourethrography (VCUG). This imaging technique allows an accurate VUR grading and detection of urethral abnormalities in male patients [1]. However, VCUG exposes children to a significant radiation dose, and in up to 50% of the cases, VUR can be missed on VCUG (“occult” VUR) while detected by isotopic cystography (IC) [2–4].

VUR constitutes a crucial risk factor for febrile urinary tract infection (fUTI) and may be associated with kidney hypo-dysplasia and, in some instances, chronic kidney disease [5, 6].

Consequently, diagnosing VUR is particularly relevant in patients with recurrent fUTIs or for correctly assessing a child with ultrasound anomalies of the kidney and urinary tract and chronic kidney disease. However, VUR diagnosis involves a non-negligible radiation dose and potentially painful procedures, necessitating optimization of radiological approaches. Taking advantage of an approach that is no longer suitable, as it exposes to an increased radiation load, we retrospectively analyzed the data deriving from a period when, in male patients, we first performed VCUG and –if negative– IC in the same session and exploiting the same catheter.

We hypothesized that patients with occult VUR exhibit similar outcomes compared to those with VCUG-detected VUR. Thus, our aim was to compare the clinical characteristics and outcomes of these two types of VUR.

## Methods

This is a retrospective study. We consecutively enrolled all male patients undergoing VCUG between January 2015 and December 2020. We selected this period because digitalized data were available, and we employed a diagnostic approach that is no longer performed, characterized by performing IC in all male patients with normal VCUG results.

Inclusion criteria were: 1) need of VCUG on the basis of the clinical history; 2) aged 1 month–17 years; 3) male sex; 4) evidence of primary VUR (absence of posterior urethral valves, PUV, and/or neurological bladder). Exclusion criteria: 1) missing clinical data; 2) refusal to undergo IC if VUR was absent on VCUG; 3) urinary tract infection (UTI) at the time of radiological investigations.

At the time of clinical management of patients, we diagnosed PUV based on direct signs of VCUG. However, drawing from our previous paper investigating direct and indirect signs of PUV [7], when retrospectively selecting patients to enroll in the present study, we excluded all patients with direct and/or indirect PUV signs on VCUG.

Our ethical committee approved the study.

### Indications to VCUG execution

VCUG was performed in case of 1) recurrent (febrile or not) UTI; 2) first fUTI by a non-Escherichia Coli organism; 3) mono- or bilateral megaureter > 7 mm of diameter; 4) mono- or bilateral hydronephrosis with an antero-posterior diameter of the pelvis (APDP) > 15 mm; 5) small kidneys (kidney length < 2 standard deviation score [SDS]) and/or mono/bilateral renal dysplasia (cortical thinning, poor corticomedullary differentiation, and renal cysts) detected with kidney ultrasound (KUS).

### Diagnostic protocol

All patients selected for this study first underwent VCUG after inserting a 5- or 6-Fr infant feeding tube into the bladder. The bladder was filled to the maximum capacity for age with a 10% solution of iodamide by drip infusion at a height of about one meter above the table. Two cycles of bladder filling and emptying were carried out [8].

In case of the absence of VUR on VCUG, during the study period, we routinely subsequently performed IC immediately after the VCUG to exploit the same bladder catheterization. The IC procedure has been previously described [2]. There was only one cycle of the bladder filling and emptying.

In accordance with our clinical practice, during follow-up of patients with VUR, when control was necessary due to recurrent fUTIs potentially indicating the need of surgical correction, all these patients were subjected to IC. Therefore, none of the enrolled patients underwent another VCUG study during the follow-up period.

### Further indications to radiological exams

All the patients newly diagnosed with VUR underwent to Tc-99m Dimercaptosuccinic acid (DMSA) scan to evaluate the presence of renal scars and to measure the reduced split renal function (SRF) [6]. In patients with fUTI/UTIs the scan was made at least 6 months after the last UTI.

### Definitions

#### Occult VUR

**Fig. 1:**
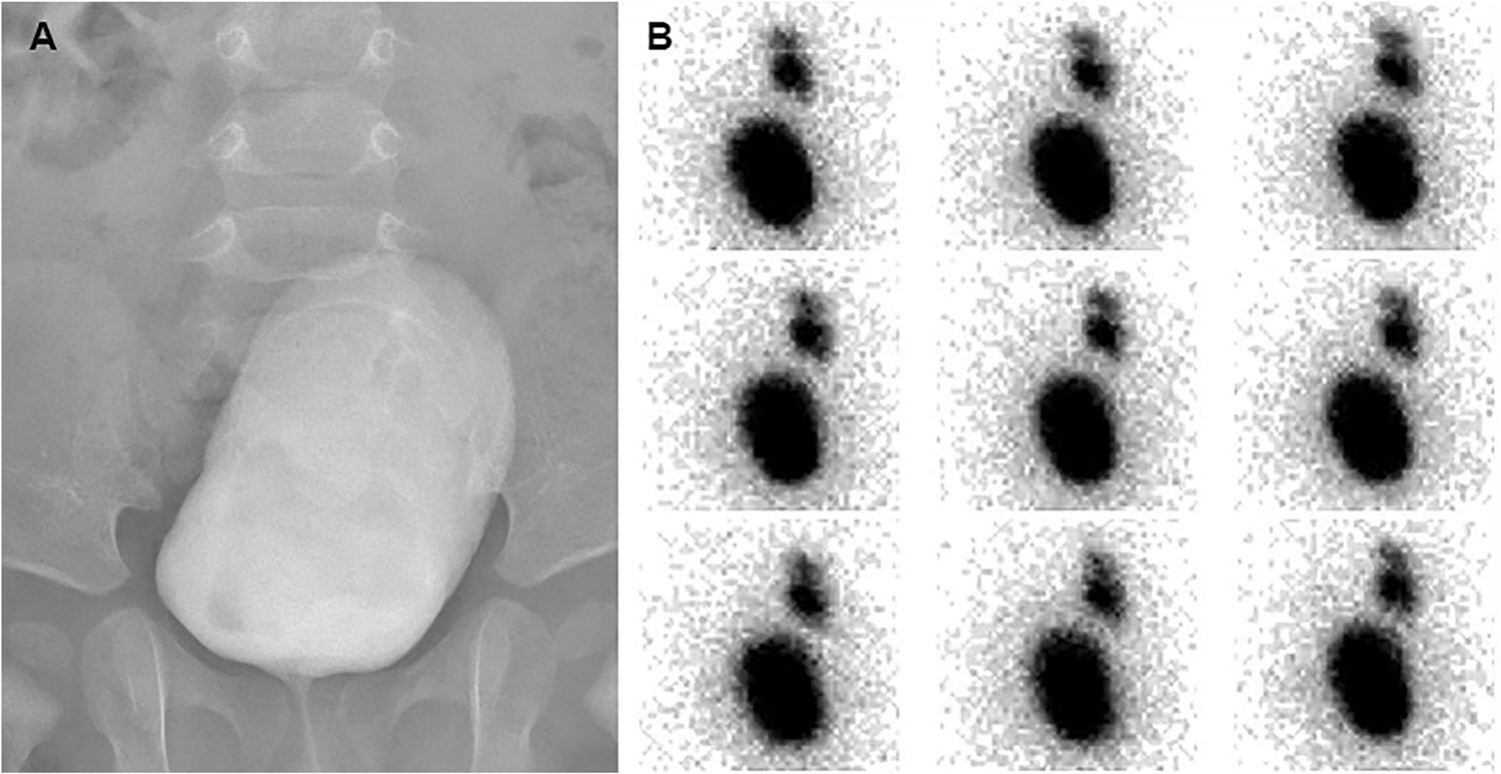
An example of occult VUR. A 4-month old boy was evaluated due to fUTI caused by E. Coli at 3 months of life. The KUS showed hydroureteronephrosis on the right side, with the anterior-posterior diameter of the pelvis measuring 16 mm and the distal ureter measuring 6 mm. VCUG (panel **A**) was negative, whereas severe VUR on the right side was detected by IC (panel **B**) performed 30 minutes later

We defined as occult VUR the presence of VUR on IC and not detected on VCUG (Fig. 1).

#### Dilated VUR

The clustering of VUR grades was adapted, classifying –on VCUG– grades III-V as “dilated” and grades I-II as “nondilated” while, on IC, severe VUR as “dilated” and mild or moderate as “nondilated”.

#### Kidney injury

Kidney injury (KI) was defined by the presence of either hypertension, proteinuria, or reduced eGFR [9]:

- hypertension was defined by the persistence of systolic (SBP) and/or diastolic (DBP) blood pressure > 95th percentile corrected for age, sex, and height.
- proteinuria was defined as persistent (confirmation within 3 months) urinary protein/urinary creatinine ratio (UPr/Cr) > 0.5 mg/mg for children < 2 years old and > 0.2 mg/mg for patients > 2 years old.
- reduced eGFR was defined by eGFR < 90 mL/min/1.73 m^2^ for children > 2 years old and according to the specific reference values for age for children < 2 years old.

In addition to a comprehensive evaluation at the time of VUR diagnosis, our patients underwent annual assessment of blood pressure and proteinuria while the serum creatinine was measured every 3 years in patients with unilateral VUR and every 2 years in patients with bilateral VUR. Serum creatinine was measured by the Jaffe method, and eGFR calculated by the original Schwartz formula [10]. This because our laboratories, at the beginning and over the duration of the study, had not yet adopted the modified assay for creatinine [11].

#### Scintigraphic damage

A SRF < 45% was defined as reduced. The presence of renal scars and/or SRF < 45% was clustered under the umbrella definition “scintigraphic damage”.

#### Febrile UTI

fUTIs were defined by the presence of urinary leukocytes and/or nitrites, a positive urine culture, and a fever > 38° C. A urine culture was considered positive if it showed ≥10^5^ colonies/mL of a single species in a sample obtained by midstream clean catch specimens or sterile bag or > 10,000 colonies/mL in samples obtained by bladder catheterization.

### Statistical analysis

We utilized the Mann-Whitney test to compare continuous variables between two groups and the Kruskal-Wallis test when comparing continuous variables among three groups, given that the analyzed variables displayed non-normal distributions. The chi-square test or Fisher exact test was used to compare the qualitative variables, when appropriate. Multiple logistic regression analysis was conducted to calculate the odds ratio (OR) of fUTI before and after VUR diagnosis and of KI for both VCUG-detected and occult VUR. We did not calculate the OR of scintigraphic damage because the patients without VUR did not undergo TC99mDMSA scan. Stat-Graph XVII software for Windows was used for all statistical analyses except for the logistic regression analysis that was performed with SPSS.

## Results

### General characteristics of the population

Four hundred and fifty-four patients were eligible for the present study. Among these, 25 were excluded for the suspect of PUV on VCUG, and 8 were excluded for denying the consent to undergo IC after VCUG. Therefore, we ultimately enrolled 421 male participants with a median age of 3 months. Sixty-one patients underwent VCUG for fUTI, with 12 experiencing recurrent fUTI. Two hundred and eleven patients underwent VCUG due to KUS abnormalities, with 124 having abnormalities other than hydronephrosis. One hundred and thirty-nine patients underwent VCUG for both indications, with 24 patients experiencing recurrent fUTI and 74 having KUS abnormalities other than hydronephrosis (Table 1). None of the patients exhibited KI at the initial evaluation, while 23 out of the 421 enrolled patients (5.4%) developed KI after a mean follow-up of 5.3 ± 4.3 years. Specifically, KI prevalence increased to 10% (20/199 patients) when separately analyzing children with VCUG-diagnosed or occult VUR. Out of the 23 patients with KI, 17 (73.9%) showed scintigraphic damage, compared to 116 out of 398 patients (29.1%) without KI (*p* < 0.001). The general characteristics of the enrolled patients are summarized in Table 1.

**Table 1:** Clinical characteristics of the enrolled patients

	General population (*n* = 421)	VCUG-diagnosed VUR (*n* = 152)	IC-diagnosed VUR (*n* = 47)	Absence of VUR (*n* = 222)	Global *p*	*p*^a^	*p*^b^	*p*^c^
Age at VUR diagnostics, median (IQR), months	3.0 (4.0)	3 (6.5)	4 (4.0)	3 (4.5)	0.32	0.99	0.24	0.35
fUTI before VUR diagnostics No. (%)	200 (47.5)	97 (63.8)	31 (66.0)	72 (32.4)	< 0.001	0.78	< 0.001	< 0.001
KUS abnormalities before VUR diagnostics, No. (%)	350 (83.1)	130 (85.5)	40 (85.1)	180 (81.1)	0.04	0.94	0.35	0.59
Both fUTI and KUS abnormalities before VUR diagnostics, No. (%)	139 (33.0)	76 (50.0)	24 (51.1)	39 (17.6)	< 0.001	0.89	< 0.001	< 0.001
Dilated VUR, No. (%)	169 (40.1)	135 (88.8)	34 (72.3)	n.a.	n.a.	0.006	n.a.	n.a.
Bilateral VUR, No. (%)	104 (24.7)	79 (52.0)	25 (53.2)	n.a.	n.a.	0.88	n.a.	n.a.
fUTI after VUR diagnostics^d^, No. (%)	60 (14.2)	41 (27.0)	10 (21.3)	10 (4.5)	< 0.001	0.43	< 0.001	< 0.001
Scintigraphic damage at last Tc99mDMSA, No. (%)	130 (30.9)	108 (71.0)	22 (46.8)	n.a	n.a	0.002	n.a	n.a
Need of surgery, No. (%)	38 (9.0)	35 (23.0)	3 (6.4)	n.a	n.a	0.01	n.a	n.a
Age at last follow-up, median (IQR), yr	4.6 (6.8)	6.0 (7.8)	4.0 (7.0)	4.1 (6.0)	0.14	0.15	0.26	0.21
SBP at the last follow-up, median (IQR), SDS	0.07 (0.9)	0.07 (0.9)	0.21 (0.96)	−0.03 (0.9)	0.20	0.08	0.82	0.10
DBP at the last follow-up, median (IQR), SDS	0.23 (0.95)	0.25 (0.87)	0.45 (0.95)	0.25 (0.9)	0.21	0.14	0.26	0.50
eGFR at last follow-up, median (IQR), mL/min/1.73 m^2^	119.0 (38.0)	117.0 (39.0)	121.0 (50.0)	119.0 (23)	0.90	0.78	0.87	0.56
UPr/Cr at last follow-up, median (IQR), mg/mg	0.17 (0.14)	0.18 (0.14)	0.15 (0.12)	0.20 (0.14)	0.35	0.36	0.30	0.19
Prevalence of KI at last follow-up, No. (%)	23 (5.4)	15 (9.9)	5 (10.6)	3 (1.3)	< 0.001	0.88	< 0.001	0.005

^a^ This *p* derives from the comparison of patients with VCUG-diagnosed VUR vs. IC-diagnosed VUR

^b^ This *p* derives from the comparison of patients with VCUG-diagnosed VUR vs. absence VUR

^c^ This *p* derives from the comparison of patients with IC-diagnosed VUR vs. absence VUR

^d^ Separately analyzing patients with dilated VUR, the prevalence of fUTI during post-VUR diagnostics follow-up was similar comparing patients with VCUG-diagnosed (38/135; 28.1%) and occult VUR (10/34; 29.4%) (*p* = 0.89)

For normal distributed variables, means ± SDS are shown, while for nonparametric ones median and interquartile range are shown

*eGFR* estimated glomerular filtration rate, *IQR* interquartile range, *n.a.* not applicable, *SBP* systolic blood pressure

### VUR diagnostics outcomes

In 222 patients (52.7%) VUR was not diagnosed either on VCUG or on IC. In 152 patients (36.1%), VUR was diagnosed on VCUG, while in 47 (11.2%), it was diagnosed on IC despite a normal VCUG. Therefore, VUR was occult in 47 out of 199 patients (23.6%) with VUR (Fig. 1). Among these 47 patients, 34 (72.3%) had dilated VUR, and 22 (46.8%) had scintigraphic damage.

### Comparison of characteristics of patients with VCUG-detected VUR, with occult VUR and without VUR (Table 1)

The age was homogeneous among groups. Patients without VUR exhibited a lower prevalence of fUTI but a similar prevalence of KUS abnormalities compared to other patients. Patients with VCUG-diagnosed VUR presented a similar prevalence of KUS abnormalities and of fUTI before VUR diagnostics compared with those with occult VUR. The coexistence of fUTI and KUS abnormalities before VUR diagnostics was more frequent among patients with VCUG-diagnosed or occult VUR compared to those without VUR.

Patients with VCUG-diagnosed VUR showed a higher prevalence of dilated VUR, a similar prevalence of fUTI during follow-up, and underwent surgical correction (endoscopic treatment or ureteral reimplantation) more frequently (23 vs. 6.4%; *p* = 0.01) compared to patients with occult VUR. Additionally, when analyzing patients with dilated VUR separately, the prevalence of fUTI during follow-up was similar (28.1 vs. 29.4%, *p* = 0.89) comparing patients with VCUG-diagnosed VUR and occult VUR.

At the last follow-up, patients with VCUG-diagnosed VUR presented a higher prevalence of scintigraphic damage compared to those with occult VUR.

Furthermore, the prevalence of KI at the last follow-up was similar between patients with VCUG-diagnosed and occult VUR, while both groups showed a significantly higher prevalence of KI compared with patients without VUR.

### Risk of fUTI and of KI (Table 2)

In the multiple logistic regression analysis, patients with VCUG-diagnosed VUR showed an increased risk of fUTI both before and after VUR diagnosis, as well as KI at the last follow-up. Conversely, patients with occult VUR showed only an increased risk of fUTI before VUR diagnosis and of KI at the last follow-up. When specifically examining patients with dilated VUR, both those with VCUG-diagnosed and occult VUR exhibited significant OR for experiencing fUTI during the post-VUR diagnostics follow-up.

**Table 2:** Multiple logistic regression analysis determining the risk of showing fUTI before and after VUR diagnostics and of showing KI in patients with VCUG- and occult VUR

	VCUG-diagnosed VUR	Occult VUR
fUTI before VUR diagnostics; OR (95%CI), *p*	3.6 (2.3–5.5); <0.001	3.9 (2.0–7.7); <0.001
fUTI after VUR diagnostics; OR (95%CI)^a^, *p*	3.1 (1.6–6.3); 0.001	2.3 (0.9–5.8); 0.08
Prevalence of KI** at last follow-up; OR (95%CI), *p*	7.9 (2.2–28.0); 0.001	8.6 (2.0–37.6); 0.004

^a^ Limiting the logistic regression analyses only to the 169 patients with dilated VUR (Table 1), the OR of developing fUTI after VUR diagnostics was—at multiple logistic regression—3.3 (95%CI: 1.6–6.7; *p* = 0.001) for VCUG-diagnosed VUR and 3.1 (95%CI: 1.2–81; *p* = 0.02) for occult VUR

** Defined by the presence of either hypertension, or proteinuria, or reduced estimated glomerular filtration rate

*CI* confidence interval

## Discussion

The data shown in this paper are no longer obtainable as they were derived from a no longer suitable approach. We adopted a diagnostic approach characterized by performing VCUG followed by IC in males when VUR was absent on VCUG. This strategy allowed us to obtain information about urethral morphology and reduced the likelihood of missing VUR. Additionally, by using the same catheter, we minimized the need for repetitive and potentially painful procedures. This approach, unfortunately, exposes children to a non-negligible radiation load totaling approximately 0.254 mSv (0.23 mSV from VCUG and 0.024 mSv from IC) [12]. Therefore, since early 2021, we no longer use this approach.

The information derived from this data, however, can be used to improve the care of patients with VUR.

The available studies on occult VUR collectively involve a total of 225 patients [2–4, 13]. The current study, involving 421 patients—approximately twice the total subjects of previous reports [2–4]—appears to give “definitive” insights into the characteristics of occult VUR in male children.

In our study, patients selected for VCUG underwent careful evaluation based on KUS findings and history of fUTI, as indirectly confirmed by the high rate of fUTI before VUR diagnostics (47.5%). The coexistence of KUS abnormalities and fUTI before VUR diagnostics was associated with both the presence of VCUG-diagnosed and occult VUR (Table 1).

We observed that approximately one-quarter of VUR cases may be missed on VCUG but identified on IC, consistent with previous findings [2–4].

The higher sensitivity of IC compared to VCUG is likely attributed to continuous monitoring of filling and voiding during the registration of scintigraphic images, which is not feasible on VCUG [2–4].

In more detail, Polito et al [2], Sukan et al [4], and Unver et al [3] found that VCUG missed VUR in 23 out of 51 patients (45.1%), 5 out of 10 patients (50%), and 4 out of 54 kidney ureter units (7.4%) with VUR, respectively. In these previous studies, the percentage of dilated VUR among the occult VUR ranged between 0 and 75% [2–4].

We found that the patients with VCUG-diagnosed VUR more frequently presented with dilated VUR and required surgical correction compared to those with occult VUR. However, patients with occult VUR also exhibited a noteworthy rate of dilated VUR (72.3%) and scintigraphic damage (46.8%) at the last follow-up. Additionally, patients with VCUG-diagnosed and occult VUR presented a similar prevalence of KI at the last follow-up. Furthermore, when restricting the analysis to patients with dilated VUR, both those with VCUG-detected and occult VUR exhibited a significantly increased risk of developing fUTI during follow-up (Table 2).

Based on our findings, it is evident that patients with VCUG-diagnosed VUR tend to have more severe VUR compared to those with occult VUR. It is also evident that patients with occult VUR exhibit a similar risk of fUTI before VUR diagnostics as during follow-up (in case of dilated VUR), and of developing KI when compared with patients with VCUG-diagnosed VUR.

We emphasize that routinely submitting patients with negative VCUG to IC is not acceptable. Nevertheless, in case of negative VCUG, clinicians should be aware that a non-negligible percentage of patients with dilated VUR and at risk of KI could be overlooked. Therefore, it may be reasonable to inform parents that a portion of VUR cases might not be detected on VCUG, but the presence of occult VUR does not necessarily warrant a different management approach. However, if a patient with negative VCUG experiences recurrent fUTIs or develops KI during the follow-up, performing an IC could be considered to assess the need for surgical correction of an occult VUR or to diagnose the etiology of a possible KI. Indirectly supporting this suggestion, Dalirani et al performed IC due to recurrent fUTI episodes in 35 children (5 males) with a mean age of 43.8 months and normal VCUG and found that 62.9% of them presented with VUR [13].

In any case, we wish to raise questions about the necessity of diagnosing VUR at any cost. To date, VCUG remains the gold standard for VUR diagnosis; however, it can potentially miss occult VUR and posterior urethral valves in a significant proportion of patients [7]. Similarly, IC may miss VUR in rare cases [2–4]. Considering that the VUR-associated nephropathy is often congenital and that the impact of fUTI on scars is limited, as is the effectiveness of antibiotic prophylaxis in reducing new scars [14], the decision to pursue VUR diagnosis in a child should primarily be guided by the evaluation of the clinical benefit derived from the radiological diagnosis. Therefore, our data should not be interpreted as a call for further studies on radiological diagnosis of VUR but rather as a means to identify patients for whom the diagnosis of occult VUR may be necessary for potential surgical intervention.

A limitation of this study is the retrospective design, and future studies analyzing less invasive approaches in VUR diagnosis and management are recommended. Another limitation is that our data are applicable only to male children.

In conclusion, occult VUR may be present in 23.6% of male children with VUR. It is of severe degree in approximately 70% of cases, with scintigraphic damage evident in about 50%. These patients also present a notable risk of developing fUTIs and KI, necessitating long-term follow-up. In male children with recurrent fUTI after a negative VCUG, IC may be justified for evaluating the need for surgical correction of a possible dilated occult VUR.

## Abbreviations

DBP: Diastolic blood pressure
eGFR: Estimated glomerular filtration rate
fUTI: Febrile urinary tract infection
IC: Isotopic cystography
KI: Kidney injury
KUS: Kidney ultrasound
OR: Odds ratio
PUV: Posterior urethral valves
SBP: Systolic blood pressure
SRF: Split renal function
UPr/Cr: Urinary protein/urinary creatinine ratio
VCUG: Voiding cystourethrogram
VUR: Vesicoureteral reflux
